# Solution Processed Trilayer Structure for High-Performance Perovskite Photodetector

**DOI:** 10.1186/s11671-018-2808-7

**Published:** 2018-12-06

**Authors:** Abbas Ahmad Khan, Zhinong Yu, Ubaid Khan, Lin Dong

**Affiliations:** 1 School of Optics and Photonics, Beijing Engineering Research Center of Mixed Reality and Advanced Display, Beijing Institute of Technology, Beijing, 100081 China; 2 School of Physics & Engineering, Zhengzhou University, Zhengzhou, 450001 China

**Keywords:** Perovskite photodetector, Solution processed, Thin film, Heterostructure, Electrical and optical properties

## Abstract

Due to their outstanding performance, low cost, ease of fabrication, diverse photonic, and optoelectronic applications, metal halides perovskite have attracted extensive interest in photodetector applications. Currently, devices made by metal oxides, metal sulfides, and 2D materials had achieved good responsivity, but suffered from high dark current, slow response speed, small on-off ratio, and poor stability. Whole performances of these photodetectors are not satisfactory. Here, a lateral perovskite (CH_3_NH_3_PbBr_3_)/Ethanolamine/TiO_2_ (in ethanol) trilayer photodetector is designed for achieving high performance. EA treatment enhances electron extraction and reduces undesired recombination. This trilayer device shows good performances with low dark current of 1.5 × 10^−11^ A, high on-off ratio of 2700, high photodetectivity of 1.51 × 10^12^ Jones, high responsivity of 0.13 A W^−1^, and high stability, comparative to conventional single layer devices. This work provides the way to improve the performance of metal halide perovskite photodetectors.

## Introduction

Photodetectors have wide range applications including optical communication, bio-medical sensing, and environmental pollution monitoring [1–3]. In the recent years, organic-inorganic lead halide perovskite materials have attracted excessive consideration due to their exceptional features such as high optical absorption, long charge carrier life time, and long diffusion length [4–9]. These characteristics suggested that the organic-inorganic perovskite are excellent materials for photodetector applications [10–21]. Up to now, the vertical structure has been widely used for photodetectors [22–27]. Recently, Zhang et al. fabricated perovskite photodetector by employing vertical structure [27]. This device demonstrated good light capability and performance, but suffered from high dark current (1.5 nA). Interestingly, lateral structures of photodetectors have attracted tremendous consideration because of low conduction losses and its simple and low-cost fabrication process. Materials such as transition metal dichalcogenides [28, 29], metal oxides [30, 31], and organic materials [32, 33] have been used for photodetector applications, among them perovskite materials attracted a lot of consideration. Single-layer perovskite devices were fabricated, but exhibited low on/off ratio, high dark current, and poor electrical instabilities [34, 35]. For example, Ding et al. fabricated a single-layer device which has high dark current, low detectivity, and low on-off ratio [35]. An organic-perovskite photodetector which is designed by Wang et al. have attained good performance, but have large dark current (ranging from 10^−7^ to 10^−8^ A) [36]. Chen et al. fabricated bilayer perovskite photodetector by introducing organic polymer layer and modified heterojunction interface to improve device performance, but still device exhibits large dark current and low detectivity [37]. However, low detectivity (D*) and high dark current is observed because of the poor heterojunction interface barrier mismatch which enhances the carrier recombination. So, suitable material choice for interfacial layer is important for device performance [33, 38]. Single crystal CH_3_NH_3_PbBr_3_ photodetectors have also been fabricated [39–41], but their results are not satisfactory because of small on-off ratio and high dark current (≈10^−10^ A).

Many approaches have been applied to improve the device performance by balancing the carriers transport and minimizing the junction resistance by matching the energy levels between the material layers. For example, metal fluorides [42], conjugated polyelectrolyte [43], and polar solvents [44] were used to reduce the energy barrier mismatch between metal oxides (ZnO, MoO_3_, ZrO_2_) and active layers to improve the optoelectronic device performance.

In this paper, we fabricated a lateral trilayer perovskite photodetector by inserting interfacial dipole layer of ethanolamine, which provides the high electron extraction with suppression of undesired carrier recombination, and as a result the device shows improved performance. In this designed structure, light with the intensity of 0.5 mW cm^−2^ absorbs in CH_3_NH_3_PbBr_3_ film and carrier’s movement is held in the alcoholic TiO_2_ film. An energy barrier gap between the alcoholic TiO_2_ and CH_3_NH_3_PbBr_3_ film is reduced by introducing ethanolamine layer. The designed trilayer photodetector shows excellent performance with low dark current of 1.5 × 10^−11^ A, photodetectivity of 1.51 × 10^12^ Jones, on-off ratio of 2700, rise time of 0.49 s, decay time of 1.17 s, linear dynamic range (LDR) of 68.6 dB, with high environmental stability.

## Results and Discussion

**Fig. 1:**
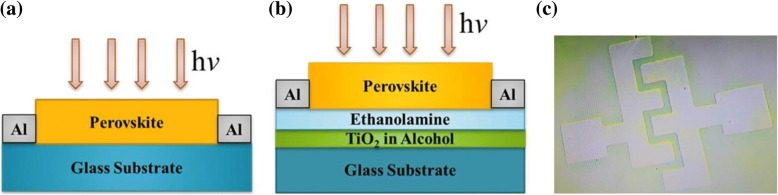
**a** The single-layer device. **b** The trilayer device. **c** Optical image of trilayer device

Single and trilayer photodetectors were fabricated on glass substrate as shown in Fig. 1a, b respectively. First of all, the TiO_2_ (mixed in ethanol) was fabricated on a glass substrate, and then ethanolamine film was deposited on the TiO_2_, afterwards, 60-nm thick Al electrodes were deposited by thermal evaporation on ethanolamine film by using shadow mask, resulting in channel width of 2000 μm and channel length of 30 μm. Then MAPbBr_3_ film was deposited on the ethanolamine (EA) film (details are in “Methods/Experimental” section). Figure 1c shows the image of trilayer photodetector.

**Fig. 2:**
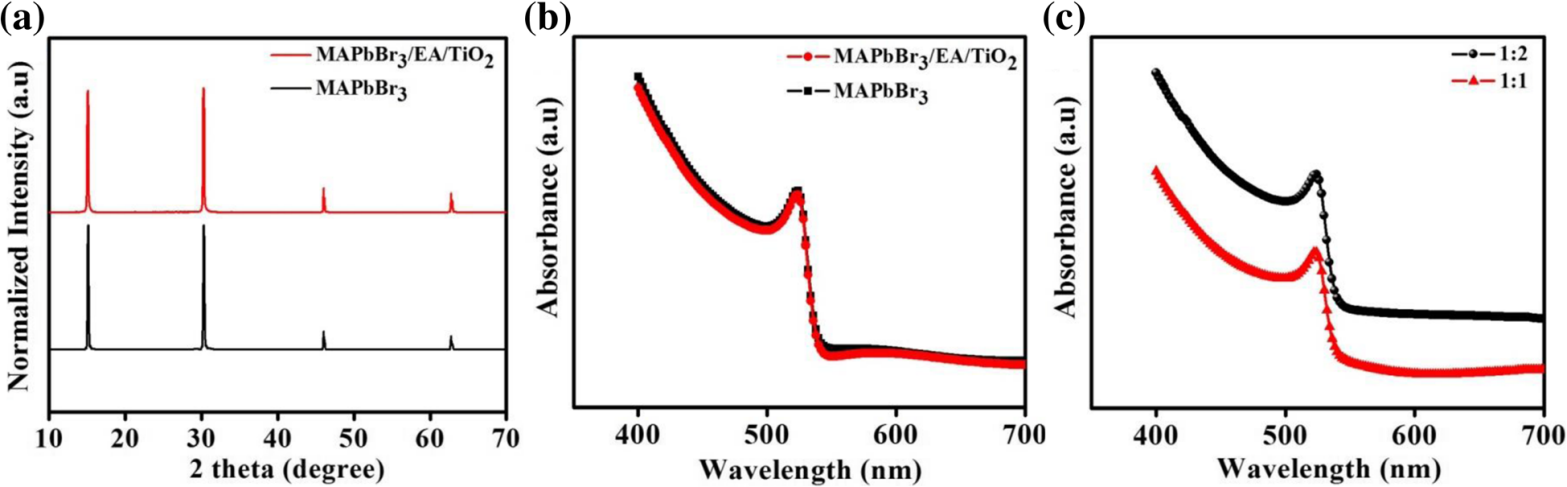
**a** X-ray diffraction patterns of the MAPbBr_3_ film and the MAPbBr_3_/EA/TiO_2_ trilayer film. **b** MAPbBr_3_ film and MAPbBr_3_/EA/TiO_2_ trilayer film absorption spectra. **c** Absorption spectra at different ratios of PbBr_2_

Figure 2a shows the XRD (X-ray diffraction) pattern of the MAPbBr_3_ film and the MAPbBr_3_/EA/TiO_2_ trilayer film. Four peaks of MAPbBr_3_ and MAPbBr_3_/EA/TiO_2_ trilayer film at 15.16°, 30.32°, 46.04°, and 62.76° are clearly observed. There are no characteristic peaks of PbBr_2_, alcoholic TiO_2_, and EA observed in MAPbBr_3_/EA/TiO_2_ trilayer film. It shows high purity of the perovskite. The Fig. 2b presents the absorption of single and trilayer films. TiO_2_ and EA films do not display any absorption. All absorption is done in perovskite film both for single and trilayer device. No prominent absorption difference is observed between the MAPbBr_3_ film and MAPbBr_3_/EA/TiO_2_ trilayer film. A band gap of 2.3 eV is also observed by absorption spectra. Absorption at different (PbBr_2_) ratios is shown in the Fig. 2c.

**Fig. 3:**
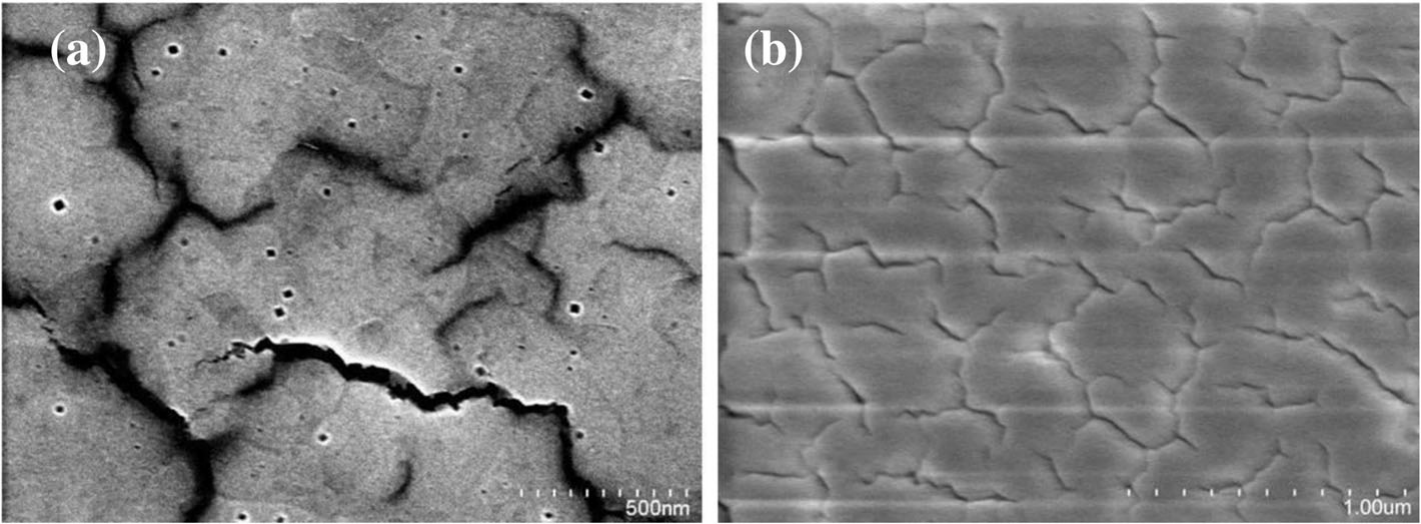
SEM image of fabricated MAPbBr_3_ film treated **a** at 100 °C and **b** at 75 °C

The surface morphology of the MAPbBr_3_ film was studied by scanning electron microscope (SEM). A large number of pin holes and cracks were observed on the perovskite film when annealed at higher temperature, as shown in Fig. 3a. Since the pin holes or cracks act as non-radiative recombination centers which results in adverse device performance [45]. On the other hand, a dense and crack-free morphology was obtained with large grain size when the perovskite film was annealed at 75 °C for 10 min (Fig. 3b). Thus, the optimized annealing temperature helped in obtaining highly crystalline films without pinholes and cracks, which can promote the separation and transportation processes of photo-excited carriers [45, 46].

**Fig. 4:**
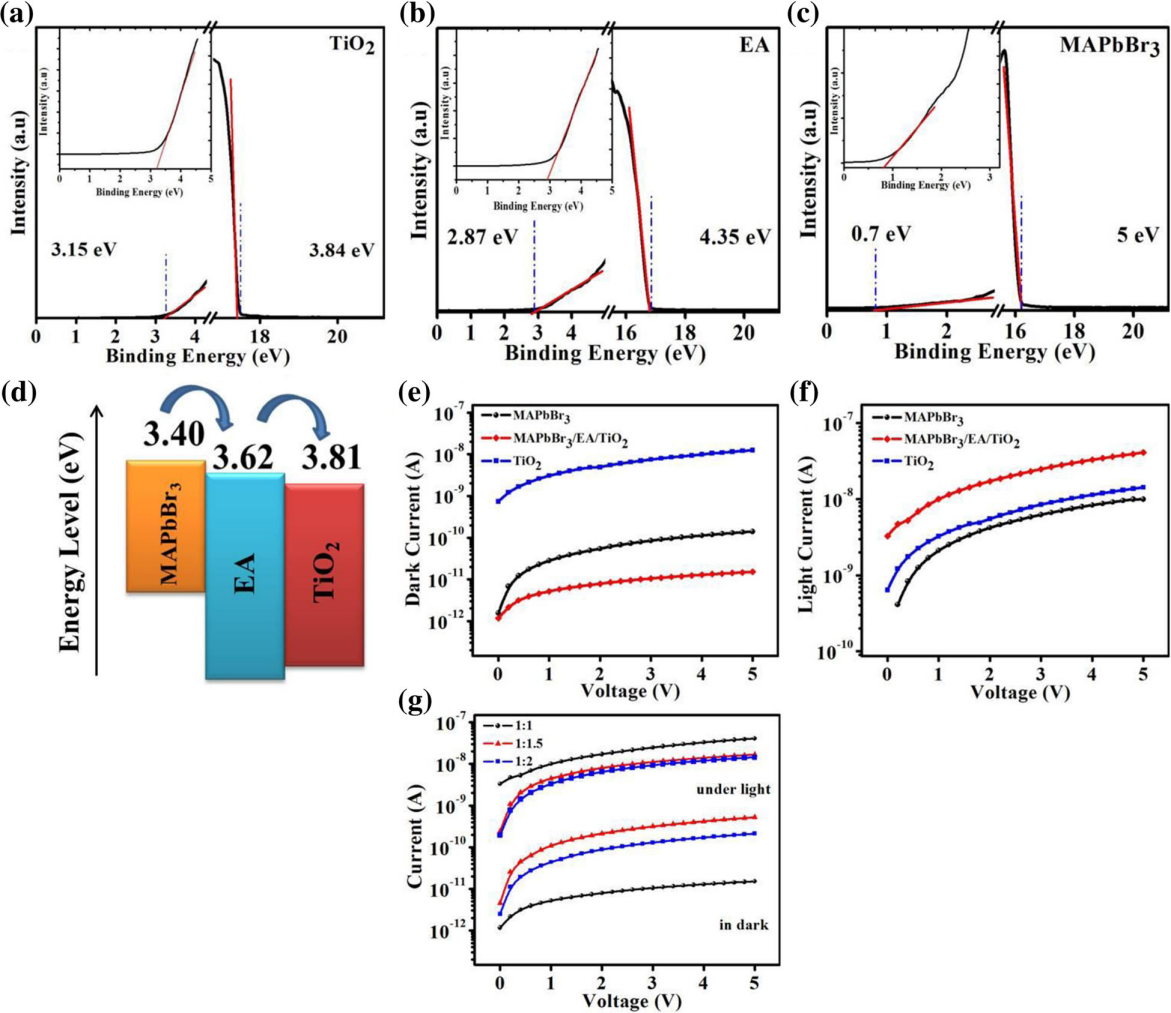
The UPS (ultraviolet photoelectron spectra) of **a** TiO_2_ film. **b** Ethanolamine film. **c** MAPbBr_3_ film. **d** Energy band diagram. *I*–*V* characteristics of photodetector devices at optimal ratio (1:1): **e** under dark, **f** under the light with intensity of 0.50 mW cm^−2^. **g** *I*–*V* characteristics of trilayer photodetector at different ratios of perovskite

Figure 4a–c shows the band energy diagrams of the alcoholic TiO_2_, ethanolamine, and the MAPbBr_3_ (determined by ultraviolet photoelectron spectroscopy (UPS) measurement). The attained Fermi levels for alcoholic TiO_2_, ethanolamine, and the perovskite are 3.84, 4.35, and 5 eV respectively. The calculated conduction band maxima (CBM) for alcoholic TiO_2_, ethanolamine, and the perovskite are 3.81, 3.62, and 3.4 eV respectively. Band energy diagram of MAPbBr_3_/EA/TiO_2_ shows that photo-generated excitons are formed in the perovskite film as shown in Fig. 4d. Therefore, electrons and holes can be segregated through the MAPbBr_3_/EA interface. Electrons will flow to EA and then fall into the TiO_2_ film, and photoholes would remain in the MAPbBr_3_ film.

Figure 4e, f presents the *I*-*V* curves of the alcoholic TiO_2_ device, MAPbBr_3_ device, and the MAPbBr_3_/EA/TiO_2_ trilayer device (under dark and under light illumination with intensity of 0.5 mW cm^−2^). A high dark current with a value of 1.24 × 10^−8^ A is calculated for alcoholic TiO_2_ device, while the value of photocurrent is nearly similar to the value of dark current. This high dark current and low photocurrent of device is observed at 5 V bias. The single-layer device fabricated by MAPbBr_3_ shows the value of dark current is 1.41 × 10^−10^ A and value of photocurrent is 9.95 × 10^−9^ A, which shows better performance than alcoholic TiO_2_-based device. In comparison, the MAPbBr_3_/EA/TiO_2_ trilayer device shows lower dark current of 1.51 × 10^−11^ A and enhanced photocurrent of 4.09 × 10^−8^ A. The depletion region created around the MAPbBr_3_/EA interface is the reason of the small amount of dark current in the MAPbBr_3_/EA/TiO_2_ trilayer device, due to which conducting areas were contracted and dark current is suppressed. A large recombination occurs in single-layer photodetectors, because of holes and electrons were transported in same layer. That is the reason photocurrent of trilayer photodetector is higher than other single-layer photodetectors. For the trilayer photodetector, photo-generated electrons and holes are separated by heterojunction. Electrons will move from MAPbBr_3_ film to the EA film and then into alcoholic TiO_2_ film through the interface. Because of this, electrons will be separated from the holes, due to which carrier recombination reduces dramatically, and this leads to a larger photocurrent. We applied different perovskite ratios to enhance the device performance as shown in Fig. 4g. The optimal perovskite ratio for trilayer photodetector is 1:1.

Responsivity (*R*) and detectivity (D^*^) are important factors for photodetector devices. Where responsivity is defined as:

1 $$ R=\frac{I_p-{I}_d}{P_{in}} $$

**Fig. 5:**
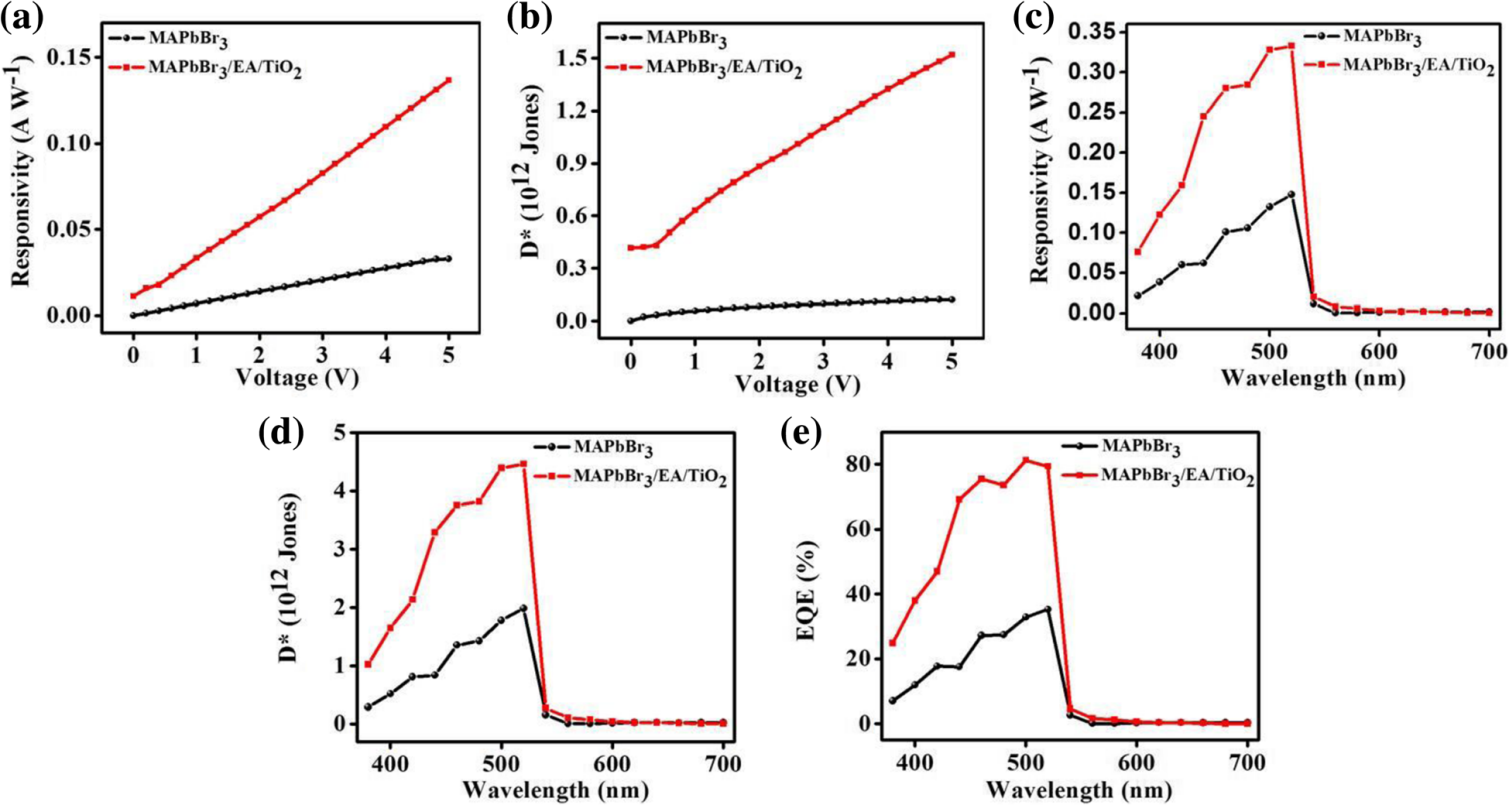
**a** Responsivity and (**b**) detectivity of single and trilayer devices at different applied voltage under light intensity of 0.5 mW cm^−2^. **c** Spectral responsivity, **d** spectral detectivity, and (**e**) EQE spectra of two photodetectors under light illumination of 10.6 μW cm^−2^ and a bias of 5 V

Where *I*_p_ represents current under white light and *I*_d_ represents current in dark. *P*_in_ represents the effective incident light power on effective region (electrode channel region) [15]. Responsivity (*R*) can be enhanced by decreasing the electrode channel length, illumination power *P*_in_, and by increasing bias voltage [30]. According to Eq. (1), responsivity for trilayer photodetector device is 0.13 A W^−1^ with incident white light intensity of 0.5 mW cm^−2^, while 0.03 A W^−1^ responsivity is calculated for single-layer photodetector device. An increased photocurrent and suppressed dark current is the reason for the enhanced responsivity of the trilayer photodetector device. Figure 5a shows the responsivity of the single and trilayer devices.

Detectivity is defined as follows:

2 $$ {D}^{\ast }=R\sqrt{\frac{S}{2q{I}_d}} $$

Where *R* is the responsivity of the photodetector device, *S* is the effective channel region under illumination, and *q* represents the electronic charge (1.6 × 10^−19^ C) [16]. Detectivity is an important parameter to signify the light sensitivity of a photodetector. Greater detectivity means larger sensitivity in detecting light signals. Figure 5b shows the detectivity of the single and trilayer devices.

According to Eq. (2), detectivity calculated for trilayer device is 1.51 × 10^12^ Jones (light intensity is 0.5 mW cm^−2^ and bias of 5 V), while value of 1.19 × 10^11^ Jones is calculated for the single-layer MAPbBr_3_ device. Trilayer device exhibits high detectivity compared to single-layer device. The very high D* of the trilayer device is due to its very low dark current.

Figure 5c, d shows the spectral responsivity and detectivity of the single and trilayer photodetector at 5 V and under light illumination of 10.6 μW cm^−2^. Trilayer device show high responsivity of 0.33 A W^−1^ and high detectivity of 4.46 × 10^12^ Jones at the wavelength of 520 nm. While for the single-layer device, responsivity is 0.14 A W^−1^ and detectivity is 1.9 × 10^12^ Jones. This shows that the trilayer photodetector can detect very weak signal of light. External quantum efficiency (EQE) spectra of both devices are measured as shown in Fig. 5e. EQE for single-layer device has been measured up to 30%, and for trilayer device up to 80% at a bias of 5 V. Absorption curve (Fig. 2b) also strengthens the results of EQE spectra.

Signal to noise ratio (SNR) and linear dynamic range (LDR) are two more important parameters to characterize a photodetector, described as:

3 $$ \mathrm{SNR}=\frac{I_p-{I}_d}{I_d} $$

4 $$ \mathrm{LDR}=20\mathit{\log}\frac{J_{\mathrm{light}}}{J_{\mathrm{dark}}} $$

Where *J*_light_ and *J*_dark_ are photocurrent and dark current density respectively [24]. The SNR can provide the detail about the level of a desired signal (photocurrent) to the background noise (dark current); background noise is less prominent when the value of SNR is high. Range of incident light power can be measured by LDR. It is generally reported in decibels (dB). LDR and SNR of the two photodetectors are measured under an applied voltage of 5 V. SNR of the single-layer device is 69. The trilayer photodetector depict a far greater SNR of 2700. The calculated LDR of trilayer photodetector is 68.6 dB, while for single-layer photodetector, LDR is 36.9 dB. The improved responsivity, detectivity, LDR, and SNR clearly shows the advantage of the trilayer photodetector over the single-layer photodetector.

**Fig. 6:**
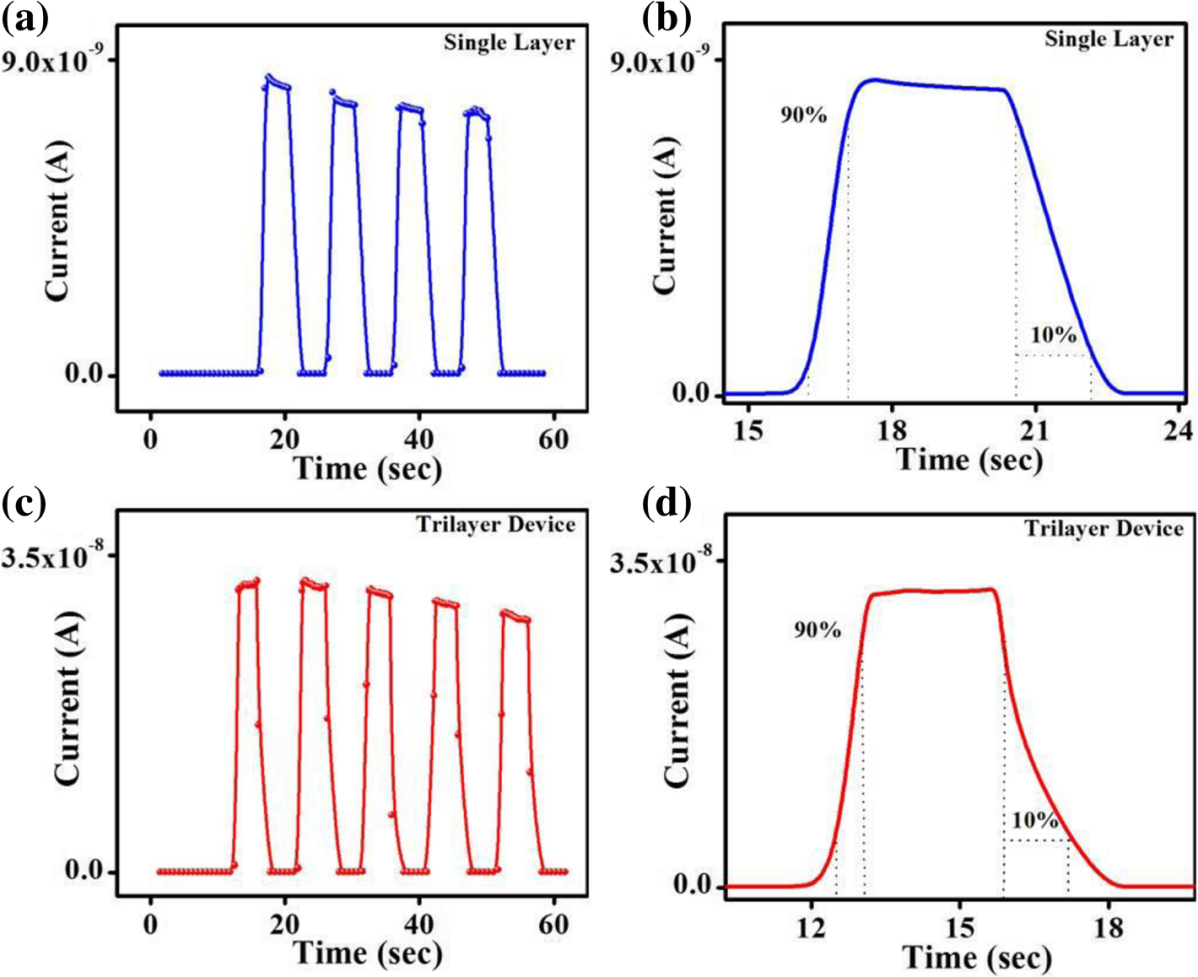
On/off switching characteristics of **a**, **b** single-layer (MAPbBr_3_) photodetector. **c**, **d** Trilayer photodetector (under light intensity of 0.5 mW cm^−2^ and applied bias of 5 V)

In Fig. 6, on/off switching characteristics of two photodetectors were measured under light intensity of 0.5 mW cm^−2^ and applied bias of 5 V. Both photodetectors exhibit excellent on-off switching repetitions, shown in Fig. 6a, c. One on-off cycle of both devices are shown in Fig. 6b, d. For single-layer device, current rises and reached to its maximum value, and then decreases slowly under illumination until the light is off. This phenomenon occurs in single-layer photodetector because the photo-generated charge carriers recombine at deep level emission sites, due to which photocurrent decreases with the passage of time. While for trilayer perovskite photodetector, current rises very quickly and reached to its maximum value, then remains stable under illumination until the light is off. This phenomenon occurs because the deep level emission sites do not affect so much photo-generated charge carriers [38]. The calculated on-off ratio was 70 for single-layer photodetector device, and measured rise/decay time is 0.72/1.72 s. While for trilayer device, on-off ratio is 2700 and its rise/decay time is 0.49/1.17 s. Rise/decay time for trilayer photodetector device is smaller comparative to single-layer photodetector device because of faster electrons and holes generation and recombination phenomenon. Time required for the photocurrent in which it reaches to 90% of its maximum value is called rise time, and time required for the photocurrent in which it drops to 10% of its maximum value is called decay time [17]. In conventional semiconductors, value of rise time and decay time is large and it originates due to long-lived traps. Separation and recombination of the charge carriers around the junction is the reason for high on-off ratio, enhanced responsivity, high detectivity, and high LDR [3, 30]. This is the reason due to which shorter rise time and decay time of trilayer photodetector device is achieved.

**Fig. 7:**
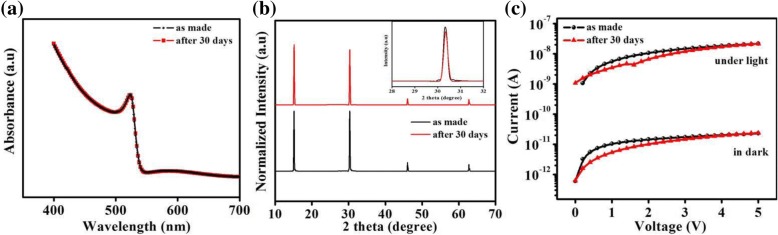
**a** Visible absorption spectra. **b** XRD spectra. **c** *I*–*V* curves of the trilayer photodetector as made and after 30 days in ambient environment (under the white light intensity of 0.5 mW cm^−2^)

Lastly, the samples were placed in an ambient environment with 30–40% relative humidity to investigate the environmental stability of the trilayer photodetector. Absorption and XRD were measured for stability as shown in Fig. 7a, b respectively. No specific changes were observed in absorption spectra after 30 days. XRD pattern were almost same after 30 days. Extra characteristic peaks due to environment were not observed. It shows the high stability of our perovskite film. The dark and photocurrent (under white light intensity of 0.5 mW cm^−2^) remain almost the same after 30 days, as shown in Fig. 7c. Trilayer photodetector device shows nearly stable photocurrent, which represents that our device is stable and less effected by ambient environment.

Overall performance with D* of 1.51 × 10^12^ Jones, on-off ratio of 2700, rise and decay time of 0.49/1.17 s, LDR of 68.6 dB, and EQE up to 80% is achieved by the trilayer device, which shows high performance of the trilayer photodetector device. Due to the excellent interface between perovskite, EA, and alcoholic TiO_2_, the excellent performance of detector is obtained. Table 1 shows the results of present metal halide perovskite photodetectors.

**Table 1:** Performance of photodetectors based on different perovskite materials

Structure	Wavelength (nm)/Power	Active area (μm^2^)	R (A W^−1^)	On/Off ratio	D (Jones)	Rise/Decay time	Ref
MAPbBr_3_	450/5 μW	400,000	0.011	–	–	2.3/2.76 s	[34]
CsPbBr_3_	450/1 mW	–	0.028	100	1.8 × 10^11^	100 ms	[35]
MAPbI_3_	365/0.13 mW 780/0.2 mW	15 × 10,000	83 5.5	324 33	2.54 × 10^13^ 1.65 × 10^12^	< 0.2 s < 0.1 s	[50]
MAPbI_3_	550/2.7 × 10^− 3^ mW	10,000 × 15,000	0.027	4.89	3.89 × 10^11^	1.2/0.2 s	[13]
CsPbBr_3_/m-TiO	350–500/150 W	10 × 2000	2	10,000	1.58 × 10^12^	> 10 s	[51]
MAPbI_3_/graphene	520/2 mW	50 × 1000	180	1.5	1 × 10^9^	0.087/0.54 s	[11]
MAPbBr_3_	570/0.4 mW	–	–	–	2 × 10^10^	1.6 ms	[21]
MAPbBr_3_	White/100 mW	–	0.1	–	7.1 × 10^11^	70/150 μs	[18]
MAPbCl_3_	365/1 W	–	0.046	1.1 × 10^3^	1.2 × 10^10^	24/62 ms	[52]
CH_3_NH_3_PbBr_3-x_I_x_	475/1 mW	–	0.055	–	–	< 20 μs	[53]
CsPbBr_3_ + Au NP	520/4.65 mW	–	0.01	1.6 × 10^6^	4.56 × 10^8^	0.2/1.2 ms	[54]
MAPbBr_2_I/graphene	405/1.05 nW	–	6 × 10^5^	1.2	> 1 × 10^14^	0.12/0.75 s	[19]
MAPbBr_3_ NW	420/ 27.5 mW	150 μm	–	61.9	–	0.12/0.08 s	[20]
MAPbI_3_/rGO	532/ 3.2 mW	12,000	73.9	23.5	–	40/28 ms	[21]
Organic/MAPbI_3_	650/0.5 mW	50 × 50	0.02	15	1.5 × 10^10^	40/140 ms	[37]
This work	White/0.5 mW	30 × 2000	0.13	2700	1.51 × 10^12^	0.49/1.17 s	–

**Fig. 8:**
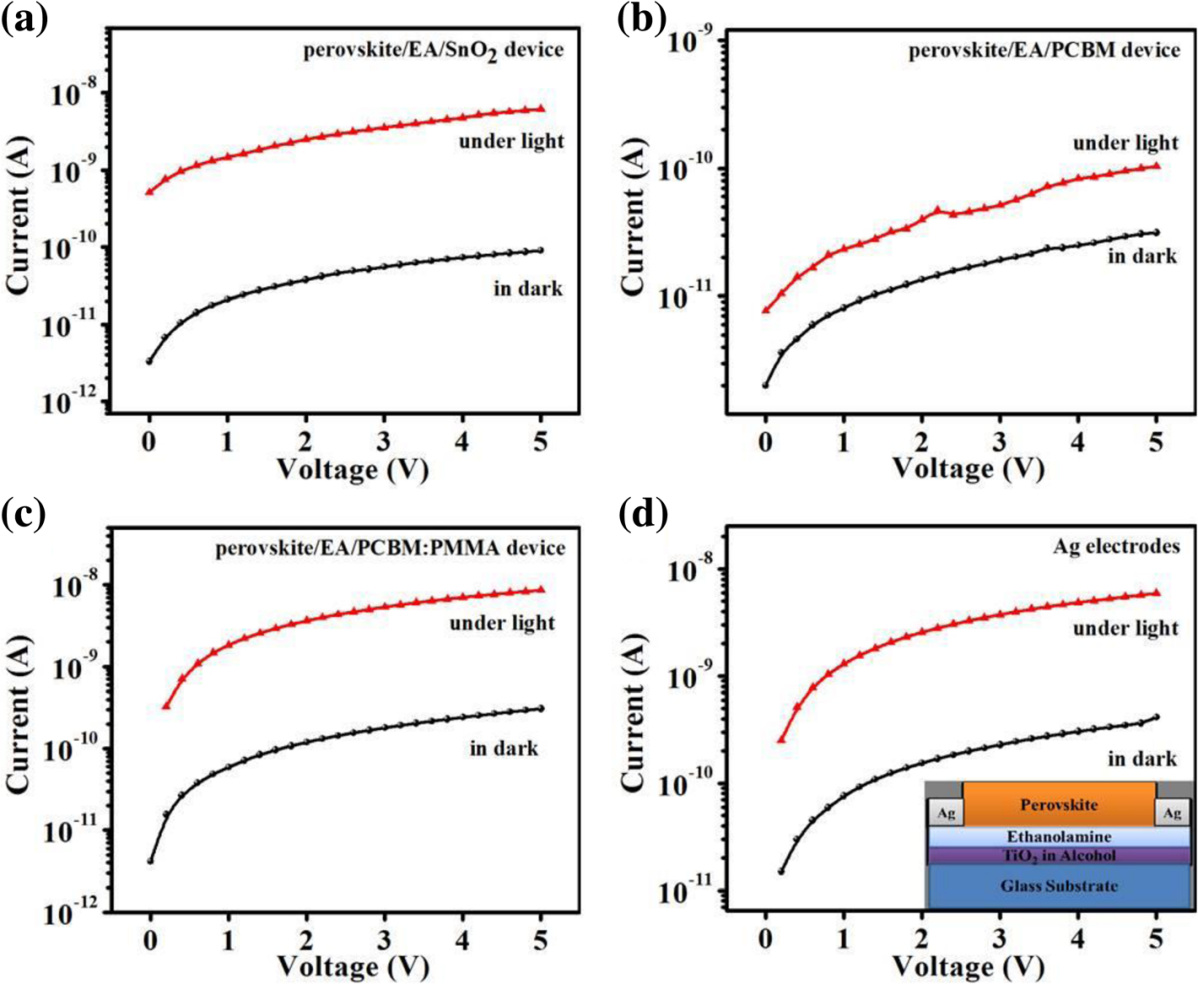
*I*–*V* curves. **a** The perovskite/EA/SnO_2_ device. **b** The perovskite/EA/PCBM device. **c** The perovskite/EA/PCBM:PMMA device. **d** The perovskite/EA/TiO_2_ device with Ag electrodes

Perovskite photodetector based on SnO_2_ was also fabricated, and due to very low on-off ratio and high dark current, the performance of the photodetector was not satisfactory as shown in Fig. 8a. All this happened due to energy level mismatching [47]. We also used phenyl-C61-butyric acid methyl ester (PCBM) and PCBM:PMMA blend as an electron transport layer to fabricated perovskite photodetector, but the performance of these devices were far much worse than SnO_2_-based devices as shown in Fig. 8b, c respectively. Choosing a suitable electron transport layer (ETL) is very important for achieving good performance of a photodetector as clearly seen by the experiments. Trilayer photodetector devices with Ag electrodes were also fabricated, but the performance of these devices was not satisfactory due to high dark current and low photocurrent as shown in Fig. 8d.

## Methods/Experimental

### Material Preparation

PbBr_2_ and CH_3_NH_3_Br were purchased from Xi’an Polymer Light Technology Corporation. DMF, DMSO, Ethanolamine, and 2-Methoxyethanol were purchased from Alfa Aesar, and TiO_2_ 10% soluble in ethanol (30 nm particle size) were purchased from InnoChem. All materials were used without additional purification. Glass slides used as substrates were manufactured by SAIL BRAND.

### TiO_2_ in Alcohol

The TiO_2_ was mixed into ethanol (ratio of 1:16 by volume) with various concentrations. A 50 μL TiO_2_ is dissolved in 800 μL ethanol and stirred for 1 h at room temperature. Due to the carriers blocking phenomenon of compact TiO_2_, it cannot be used as a good electron transport layer [48]. So that, TiO_2_ mixed in alcohol was used as ETL.

### Ethanolamine

EA and 2-methoxyethanol mixture were synthesized by using previously reported method [49]. The ethanolamine 3%wt ratios was mixed into 2-methoxyethanol to prepare solution mixture.

### Perovskite

A 1 M solution of CH_3_NH_3_PbBr_3_ was prepared by reacting the CH_3_NH_3_Br and PbBr_2_ at a 1:1 (by weight) in a mixture of dimethylsulfoxide (DMSO) and *N*,*N*-dimethylformamide (DMF) with 1:4 (by volume), then stirring was applied at the temperature of 70 °C for overnight.

### Device Fabrication

Glass substrates were washed with detergent, deionized water, isopropyl alcohol, and acetone solvents for 20 min each and then dried with N_2_ air, and were finally cleaned by O_2_ plasma for 15 min to eliminate the particles left behind on the substrates. Firstly, glass substrates were spin coated by alcoholic TiO_2_ at the speed of 4000 rpm for 30 s under ambient environment, and then annealed at 150 °C for 30 min. Afterwards, EA in 2-methoxyethanol was deposited on the TiO_2_ film at the speed of 3000 rpm for 40 s under ambient environment, and then annealed at 130 °C for 10 min. Then, Al (aluminum) electrodes with the thickness of 60 nm were deposited on EA film by thermal evaporation. The channel width of the shadow mask is 2000 μm and the channel length of the shadow mask is 30 μm. Finally, MAPbBr_3_ solution was spin coated on the EA film by two-step method to complete the fabrication process. In the first step, solution was spin coated with the speed of 1000 rpm for 10 s, and then spin coated with the speed of 5000 rpm for 30 s. During the second step, 50 μL of toluene were dropped on the spinning substrate at 22 s before the spin coating end.

### Characterization

Keithley 4200 was used to measure the electrical characterizations under ambient environment at room temperature. XZ-150WA cold light illuminator was used as a source of white light. Before using the white light, the intensity of the light was measured by monosilicon detector. Newport Oriel 200 was used as a source of monochromatic light. SEM (Hitachi S-4800) was used to characterize the surface image and morphology of the film. JASCO V-570 spectrophotometer was used to record the absorption spectra. KRATOS AXIS ULTRA DLD photoelectron spectroscopy system with an unfiltered He I (21.22 eV) gas-discharge lamp was used to record the UPS analysis. Rifaku D/MAX-2004 XRD with Cu *K* radiation ($\lambda =1.54178\ \mathrm{\AA}$) was used to study the phase identification of the film, which is operating at 60 mA and 40 kV.

## Conclusions

In conclusion, a perovskite/EA/TiO_2_ trilayer photodetector was designed and fabricated. The dark current is significantly reduced in trilayer photodetector device because of formed heterojunction. The EA film and the alcoholic TiO_2_ film were used to fabricate perovskite/EA/TiO_2_ trilayer photodetector device. In this type of design, light absorbs in perovskite film, and photoelectrons transported in alcoholic TiO_2_ film, and EA is responsible for decreasing the energy barrier mismatch and enhances the photoelectron extraction. When light is illuminated, charge carriers are separated through the heterojunction. The electrons are transferred into EA layer then transferred and transported in to alcoholic TiO_2_, and the holes remains in the perovskite layer. As a result, charge carrier’s recombination suppressed and photocurrent enhances. The overall performance of trilayer device shows D* of 1.51 × 10^12^ Jones, on-off ratio of 2700, *R* of 0.13 A W^−1^, rise and decay time of 0.49/1.17 s, and LDR of 68.6 dB. The MAPbBr_3_/EA/TiO_2_ photodetector device shows very high whole performance as compare to single crystal-based devices and 2D material-based devices. The stability of the trilayer device in an ambient environment shows high significance in future optoelectronics devices. A modified interfacial layer and electron transport layer can significantly suppress the carrier recombination and improve the performance of perovskite photodetector devices. This approach could play an important role to improve device performance by heterojunction modifications.

## Abbreviations

EA: Ethanolamine
EQE: External quantum efficiency
LDR: Linear dynamic range
PCBM: Phenyl-C61-butyric acid methyl ester
PMMA: Polymethylmethacrylate
SEM: Scanning electron microscope
SNR: Signal to noise ratio
UPS: Ultraviolet photoelectron spectroscopy
XRD: X-ray diffraction
